# Interaction with adenylate cyclase toxin from *Bordetella pertussis* affects the metal binding properties of calmodulin

**DOI:** 10.1002/2211-5463.12138

**Published:** 2016-12-09

**Authors:** Tzvia I. Springer, Corey C. Emerson, Christian W. Johns, Natosha L. Finley

**Affiliations:** ^1^ Department of MicrobiologyMiami UniversityOxfordOHUSA; ^2^ Cell, Molecular, and Structural Biology ProgramMiami UniversityOxfordOHUSA; ^3^ Present address: Department of PharmacologyCleveland Center for Membrane and Structural BiologyCase Western Reserve UniversityClevelandOH44106USA

**Keywords:** adenylate cyclase, calcium, calmodulin, magnesium, nuclear magnetic resonance

## Abstract

Adenylate cyclase toxin domain (CyaA‐ACD) is a calmodulin (CaM)‐dependent adenylate cyclase involved in *Bordetella pertussis* pathogenesis. Calcium (Ca^2+^) and magnesium (Mg^2+^) concentrations impact CaM‐dependent CyaA‐ACD activation, but the structural mechanisms remain unclear. In this study, NMR, dynamic light scattering, and native PAGE were used to probe Mg^2+^‐induced transitions in CaM's conformation in the presence of CyaA‐ACD. Mg^2+^ binding was localized to sites I and II, while sites III and IV remained Ca^2+^ loaded when CaM was bound to CyaA‐ACD. 2Mg^2+^/2Ca^2+^‐loaded CaM/CyaA‐ACD was elongated, whereas mutation of site I altered global complex conformation. These data suggest that CyaA‐ACD interaction moderates CaM's Ca^2+^‐ and Mg^2+^‐binding capabilities, which may contribute to pathobiology.

**Abbreviations**
Ca^2+^: calcium
CaM: calmodulin
CyaA‐ACD: adenylate cyclase toxin domain
DLS: dynamic light scattering
Mg^2+^: magnesium

*Bordetella pertussis* secretes an adenylate cyclase toxin (CyaA), a critical bacterial virulence factor, which is activated by calmodulin (CaM) during host cell infection. CyaA is composed of a 141 kDa hemolytic C‐terminal domain and a 43 kDa N‐terminal adenylate cyclase toxin domain (CyaA‐ACD) 1, 2 When stimulated by CaM, CyaA‐ACD converts ATP to cAMP leading to a pathophysiological accumulation of intracellular cAMP 3, 4, 5. Interaction with both N‐terminal and C‐terminal domains of intact CaM contributes to a 400‐fold increase in CyaA‐ACD binding affinity as compared to the isolated N‐terminal or C‐terminal domain, but the molecular mechanism remains to be determined 6. *In vitro* assays demonstrate that CaM‐dependent CyaA‐ACD activation occurs at low calcium (Ca^2+^) (0.1 μm) in the presence of high magnesium (Mg^2+^) concentrations (0.1–10 mm) 6, conditions under which the metal binding characteristics of these proteins likely contribute to catalysis. Moreover, elevated Ca^2+^ inhibits CyaA‐ACD activation, necessitating increased Mg^2+^ concentration for stimulation, but the metal‐dependent mechanisms and the roles that CyaA‐ACD and CaM play in this process remain to be determined.

Calmodulin is a ubiquitous eukaryotic protein that senses changes in cytosolic Ca^2+^ levels in response to physiological stimuli. As a member of the EF‐hand family of proteins, CaM and other Ca^2+^‐responsive proteins, such as troponin C (TnC), have helix‐loop‐helix structural motifs that ligate Ca^2+^ in response to cellular signaling 7. In the cell, resting levels of intracellular free Mg^2+^ are maintained in the millimolar range, while Ca^2+^ concentrations fluctuate from 0.1 to 0.5 μm upon stimulation. In order to transmit the signals involved in triggering Ca^2+^‐regulated biological processes, CaM must differentially bind Ca^2+^ and Mg^2+^.

Calmodulin has four highly conserved Ca^2+^/Mg^2+^‐binding sites. It is composed of N‐terminal and C‐terminal globular domains connected by a flexible tether. Sites I and II are in the N‐terminal domain while sites III and IV are located in the C‐terminal domain of CaM. It is known that both domains of CaM cooperatively bind Ca^2+^ with higher affinity than Mg^2+^. The C‐terminal domain, which is known to play a prominent role in target binding, has higher Ca^2+^ affinity than the N‐terminal domain. In contrast, the N‐terminal domain binds Mg^2+^ with higher affinity than the C‐terminal domain, indicating that preferential metal binding in each domain may have regulatory functions in CaM‐dependent activation. In the absence of Ca^2+^‐coordination, the globular domains of CaM are ‘closed’ (ApoCaM) with minimal exposure of the hydrophobic clefts. Ca^2+^ binding to sites I–IV promotes conformational rearrangements in CaM and subsequent exposure of hydrophobic surface areas in each domain, which is important in target peptide recognition 8. While Mg^2+^ binding induces smaller, yet distinct structural transitions in CaM 9, 10, 11, 12, it is postulated to serve mainly as a suppressor of CaM‐dependent activation. In the presence of physiologically relevant Mg^2+^ concentrations, it is reported that Ca^2+^ affinity in CaM diminishes. However, the Mg^2+^‐binding constants in CaM are such that it is likely to be fully or partially Mg^2+^ loaded at resting Ca^2+^ concentrations 12. The probability of multiple intracellular populations consisting of Mg^2+^/Ca^2+^‐loaded CaM conformers would suggest that a simple ‘on/off’ regulatory switch may not be adequate to control CaM‐dependent enzyme activation.

Although the molecular mechanisms are unknown, Ca^2+^‐independent and ‐dependent activation of CyaA‐ACD occurs upon interaction with CaM 13. To date, there is no high‐resolution structure of intact CaM bound to CyaA‐ACD, so the role of metal binding in the catalytic stimulation of this system remains obscure. A functionally homologous adenylate cyclase toxin made by *Bacillus anthracis* (EF) engages the N‐terminal domain of CaM through extensive intermolecular contacts with the helical domain. However, N‐terminal CaM remains in a closed conformational state when associating with EF and requires elevated Ca^2+^ for activation 14, 15, 16. In direct contrast, CaM‐dependent activation of CyaA‐ACD occurs at lower Ca^2+^ concentrations and is not regulated by intracellular Ca^2+^ concentration 6, 17, 18. N‐terminal CaM is required for full activation even though CaM mutants defective in N‐terminal domain opening also activate CyaA‐ACD 6. The N‐terminal domain of CaM has a unique conformational state upon CyaA‐ACD association that does not resemble ‘closed’ CaM 19. Furthermore, we showed that N‐terminal CaM directly contacts CyaA‐ACD through interactions involving the β‐hairpin of CyaA‐ACD. Abolishing the intermolecular association between N‐terminal CaM and the β‐hairpin promotes conformational exchange in metal‐binding site II of CaM 19, 20. Clearly, interaction with N‐terminal CaM, and metal‐induced conformational transitions in CaM, are important factors in controlling CyaA‐ACD association, but the structural mechanisms remain unclear. Taken together, the collective evidence points to the possibility that bacterial adenylate cyclase toxins, in particular CyaA‐ACD, have evolved novel modes of activation from their eukaryotic counterparts. Exploiting these molecular differences in CaM‐dependent adenylate cyclase activation may provide a means by which to develop highly specific, novel therapeutic agents to combat emerging infectious diseases. In our present study, we seek to examine the roles of Mg^2+^ and Ca^2+^ binding in CaM/CyaA‐ACD complex formation using NMR, dynamic light scattering (DLS), site‐directed mutagenesis, and native PAGE. These findings provide a structural framework from which to consider the potential role Mg^2+^/Ca^2+^ binding has in CaM‐dependent enzyme activation.

## Materials and methods

### Sample preparation

Recombinant CyaA‐ACD was overproduced and purified as previously described 19. Cell pellets containing insoluble CyaA‐ACD were resuspended on ice in buffer containing 8 m Urea, 500 mm NaCl, 40 mm imidazole, 20 mm Tris‐HCl, and 1 mm PMSF at pH 8.0 and lysed by sonication. CyaA‐ACD was resolved using HisTrapTM HP Ni‐Sepharose resin (GE Healthcare, Pittsburgh, PA, USA). Recombinant CyaA‐ACD was eluted from columns with increasing concentrations of imidazole and identified as homogenous as analyzed by SDS/PAGE, as previously described 19. Bradford assay was used to quantify purified CyaA‐ACD.

Recombinant stable isotope labeled and unlabeled CaM were expressed, purified, and quantified as previously described 19. Site‐directed mutagenesis of Glu31Asp in CaM (CaME31D) was performed according to the manufacturer's protocol using the Quikchange^™^ mutagenesis kit (Santa Clara, CA, USA). Recombinant CaME31D was also expressed, purified, and quantified as previously described 19. All CaM samples were concentrated using a centricon (Corning, Oneonta, NY, USA) and stored at −20 °C.

ApoCaM was prepared by dialyzing purified recombinant proteins against 4 L of buffer containing 250 mm NaCl, 20 mm EDTA, 20 mm EGTA, 20 mm Hepes‐NaOH pH 7.3, and 1 mm PMSF. Fully Mg^2+^‐loaded CaM (4Mg^2+^‐CaM) was prepared by dialysis and ultracentricon buffer exchange of ApoCaM into 250 mm NaCl, 40 mm MgCl_2_, 20 mm Hepes‐NaOH pH 7.3, and 1 mm PMSF. Partially 2Mg^2+^/2Ca^2+^‐loaded CaM (2Mg^2+^/2Ca^2+^‐CaM) was prepared by dialyzing 4Ca^2+^‐CaM against 250 mm NaCl, 40 mm MgCl_2_, 20 mm Hepes‐NaOH pH 7.3, and 1 mm PMSF. For all experiments, ultrapure MgCl_2_ and CaCl_2_ were used. Complex formation between CyaA‐ACD and stable isotope‐labeled CaM was performed as previously described 19.

For NMR analyses, samples of [^2^H, ^15^N] ApoCaM or [^2^H, ^15^N, ^13^C] ApoCaM ranging in protein concentration between 0.5 and 0.7 mm were suspended in 250 mm NaCl, 1 mm EGTA, 40 mm MgCl_2,_ 20 mm Hepes‐NaOH pH 7.3, 1 mm PMSF, and 10%D_2_O (NMR buffer). To prepare samples of recombinant 2Mg^2+^/2Ca^2+^‐CaM and 2Mg^2+^/2Ca^2+^‐CaM/CyaA‐ACD, purified proteins consisting of [^2^H, ^15^N, ^13^C] 4Ca^2+^‐CaM or [^2^H, ^15^N, ^13^C]4Ca^2+^‐CaM/CyaA‐ACD were exchanged into buffer containing 40 mm MgCl_2_ using dialysis followed by centricon concentration into NMR buffer.

### NMR spectroscopy

NMR experiments were performed on a Bruker Avance III (www.bruker.com) 600 MHz spectrometer equipped with a conventional 5‐mm probe. Two dimensional Transverse Relaxation Optimized (^1^H‐^15^N TROSY‐HSQC) spectra were collected for all samples at 298 K. The metal occupancy of each sample was monitored by 2D TROSY‐HSQC as each state had a unique spectrum similar to previously published data 19, 21, 22. The backbone chemical shift assignments for [^2^H, ^15^N, ^13^C]2Mg^2+^/2Ca^2+^‐CaM/CyaA were determined in part by direct comparison to the previously known values of [^2^H, ^15^N, ^13^C]4Ca^2+^‐CaM/CyaA‐ACD and confirmed by using the following suite of triple‐resonance experiments: ^15^N edited NOESY‐HSQC, TROSY‐HNCO, TROSY‐HNHA, and TROSY‐HNCA. NMR data were processed using NMRPipe 23 analyzed using sparky 24. Amide proton‐nitrogen chemical shift perturbations were calculated as described 19.

### Dynamic light scattering experiments

Dynamic light scattering measurements were performed on 20 μm samples suspended in NMR buffer (containing 10 mm CaCl_2_ or 40 mm MgCl_2_) using the Malvern Zetasizer Instrument (Malvern, Westborough, MA, USA) equipped with a temperature controller. Prior to recording DLS data, samples were centrifuged and filtered to remove particulate matter. Aliquots of 90 μL were dispensed into the small volume cuvette and measurements were collected for 4Ca^2+^‐CaM, 4Ca^2+^‐CaM/CyaA‐ACD, 2Mg^2+^/2Ca^2+^‐CaM/CyaA, and 2Mg^2+^/2Ca^2+^‐CaME31D/CyaA‐ACD complexes. Data were analyzed using Malvern zetasizer software (Malvern).

### Native PAGE

Routinely, samples were analyzed following complex formation and size exclusion chromatography (SEC) by native PAGE. For small‐scale complex formation, increasing amounts of CyaA‐ACD (0–2.5 μm) were titrated into samples of 5 μm CaM or CaM(E31D) in the presence of 250 mm NaCl, 20 mm Hepes‐NaOH pH 7.3, and 1 mm PMSF buffer containing either 10 mm CaCl_2_ or 40 mm MgCl_2,_ respectively. Following incubation at room temperature, samples consisting of free CaM, free CyaA, and CaM/CyaA‐ACD complexes were subjected to native PAGE in the presence of 10 mm CaCl_2_ or 40 mm MgCl_2_ at 4°C. Proteins were visualized by Coomassie Brilliant Blue staining.

## Results and Discussion

### Metal binding in N‐terminal CaM upon CyaA‐ACD association

**Figure 1:**
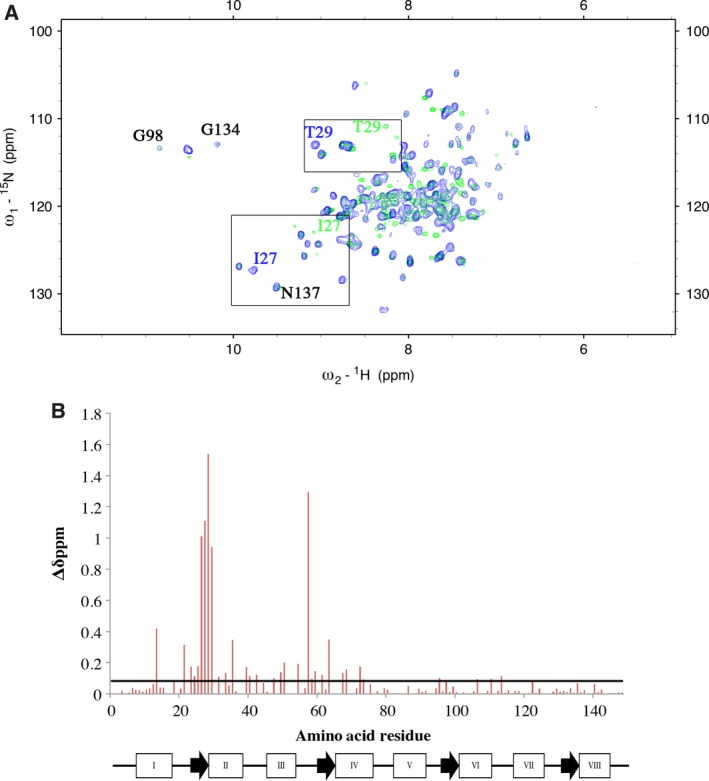
Comparison of 2D ^1^H‐^15^N TROSY spectra revealing that Mg^2+^ binding is localized to sites I and II in the CaM/CyaA‐ACD complex. (A) [^15^N, ^13^C, ^2^H]‐CaM bound to CyaA‐ACD in the presence of 4Ca^2+^ saturation (blue) and 2Mg^2+^/2Ca^2+^ saturation (green). Selected residues are labeled according to the amino acid residue. (B) Amide proton‐nitrogen chemical shift differences mapping to residues in [^15^N, ^13^C, ^2^H]‐CaM/CyaA‐ACD that are perturbed by Mg^2+^ binding to sites I and II. The secondary structure of CaM is summarized with rectangles (helices) and arrows (β‐sheets). The average chemical shift change + one standard deviation is indicated by the horizontal black line. The observation of overlapping resonances mapping to the C‐terminal domain of CaM in both the 4Ca^2+^‐CaM‐CyaA‐ACD and 2Mg^2+^/2Ca^2+^‐CaM/CyaA‐ACD complexes indicates that sites III and IV remain Ca^2+^ loaded. In contrast, the amide proton‐nitrogen resonances in the N‐terminal domain have Mg^2+^‐specific conformations in the 2Mg^2+^/2Ca^2+^‐CaM/CyaA‐ACD complex.

To investigate the influence of CyaA‐ACD association on the metal binding properties of CaM, we monitored Mg^2+^‐induced structural changes in this system by solution NMR, DLS, and native PAGE. We have previously shown that these tools are useful for probing CyaA‐ACD‐induced conformational transitions in 4Ca^2+^‐CaM 19. In the present study, stable isotope labeled CaM was used to generate complexes with unlabeled CyaA‐ACD in the presence of Mg^2+^. Under these conditions, it was determined that the N‐terminal domain of CaM is Mg^2+^ loaded in the presence of CyaA‐ACD. Given that we were able to clearly distinguish the various metal‐ligated states of CaM in the presence and absence of CyaA‐ACD, we reasoned that NMR would permit the observation of conformational changes occurring at each amino acid ligand. The global conformations of 4Ca^2+^‐CaM/CyaA‐ACD and 2Mg^2+^/2Ca^2+^‐CaM/CyaA‐ACD were similar as evidenced by comparison of 2D ^1^H‐^15^N TROSY spectra (Fig. 1). Association of CyaA‐ACD with CaM occurred in slow exchange on the NMR time scale which permits the determination of resonance assignments for ≥ 85% of the residues in CyaA‐ACD‐bound CaM complexes. The availability of chemical shift assignments for N‐terminal 2Mg^2+^‐CaM facilitated resonance assignment for 4Mg^2+^‐CaM 22. The chemical shift assignments of free 2Mg^2+^/2Ca^2+^‐CaM were also based in part on published values. However, significant exchange broadening of residues mapping to the metal‐binding sites of CaM was observed for 2Mg^2+^/2Ca^2+^‐CaM, most likely a consequence of exchange between ApoCaM and Mg^2+^‐loaded conformers. Upon interaction with CyaA‐ACD, Ca^2+^ affinity in C‐terminal CaM was increased and, it was possible to monitor Mg^2+^ binding to N‐terminal CaM in the 2Mg^2+^/2Ca^2+^‐CaM/CyaA‐ACD complex. Routinely, it was feasible to directly transfer many of the chemical shift assignments from 4Ca^2+^‐CaM/CyaA‐ACD to 2Mg^2+^/2Ca^2+^‐CaM/CyaA‐ACD. Verification of sequential resonance assignments was achieved using ^15^N‐edited NOESY‐HSQC experiments collected at 70‐ and 150‐ms mixing times. The backbone Cα and C′ resonances were correlated with ^1^H and ^15^N amide residues using multidimensional, heteronuclear NMR experiments. Observed backbone chemical shift values were similar to 4Ca^2+^‐CaM/CyaA‐ACD, indicating that Mg^2+^ binding does not significantly alter the secondary structure of CaM in the presence of CyaA‐ACD.

Comparison of the ^1^H‐^15^N TROSY spectra of [^2^H, ^15^N, ^13^C]2Mg^2+^/2Ca^2+^‐CaM/CyaA and [^2^H, ^15^N, ^13^C]4Ca^2+^‐CaM/CyaA‐ACD revealed Mg^2+^ binding is limited to N‐terminal CaM in the presence of CyaA‐ACD (Fig. 1). For example, the amide proton‐nitrogen resonances for T29 and I27, mapping to site I in N‐terminal CaM, exhibited metal‐specific conformations in the 2Mg^2+^/2Ca^2+^‐CaM/CyaA‐ACD and 4Ca^2+^‐CaM/CyaA‐ACD complexes (Fig. 1A). NMR chemical shift mapping revealed that Mg^2+^ binding is localized to sites I and II in the 2Mg^2+^/2Ca^2+^‐loaded complex (Fig. 1B). The residues involved in Mg^2+^ ligation and their corresponding chemical shift values were similar to those reported for free N‐terminal CaM 22. It has been shown that the smaller ionic radius of the Mg^2+^ ion has different coordination geometry than the larger Ca^2+^, which limits the conformation plasticity in EF‐hand proteins 12, 22. Additionally, we found that residues mapping to sites I and II were perturbed in a way that is indicative of Mg^2+^ ion coordination at each metal‐binding loop. It is known that NMR chemical shift values of residues at positions 8 of the metal‐binding loops in Ca^2+^‐ or Mg^2+^‐loaded CaM are useful indicators of the metal occupancy 22, 25. Consistent with this, the amide nitrogen chemical shift differences at positions I27 and I63 in 2Mg^2+^/2Ca^2+^‐CaM/CyaA‐ACD were shifted +11.0 and +4.1 p.p.m., respectively, compared to ApoCaM (data not shown). The magnitude of the amide chemical shift differences were less substantial at positions 8 in site I and II of 2Mg^2+^/2Ca^2+^‐CaM/CyaA‐ACD than that observed in the fully loaded Ca^2+^ complex which likely reflects that CyaA‐association promotes a less ‘open’ conformational state in N‐terminal CaM. These findings are indicative of CaM being a structurally dynamic Ca^2+^‐sensing protein, a property which is known to function in metal binding and target recognition 26, 27, 28. Taken together, these data support that 2Mg^2+^ ions are bound to N‐terminal CaM in the presence of CyaA‐ACD.

In contrast, sites III and IV in 2Mg^2+^/2Ca^2+^‐CaM/CyaA‐ACD were not affected by the presence of Mg^2+^, showing that Ca^2+^ remains bound to C‐terminal CaM. Resonances G98 and N137 mapping to the C‐terminal CaM were overlapped in the TROSY spectra of 2Mg^2+^/2Ca^2+^‐CaM/CyaA‐ACD and 4Ca^2+^‐CaM/CyaA‐ACD complexes, which suggests that the C‐terminal domain remains Ca^2+^ loaded in these complexes (Fig. 1A). No detectable conformational changes were observed in the C‐terminal domain as indicated by the lack of significant amide proton‐nitrogen chemical shift perturbations (Fig. 1B). This strongly supports that CyaA‐ACD association prevents Ca^2+^/Mg^2+^ exchange in C‐terminal CaM by increasing Ca^2+^ affinity. Even in the presence of excess Mg^2+^ concentrations, Ca^2+^ remained bound to sites III and IV in the 2Mg^2+^/2Ca^2+^‐loaded CaM/CyaA‐ACD complex. These findings are significant because they point to the possibility that even in the resting state, when cytosolic Ca^2+^ concentrations are low, 2Mg^2+^/2Ca^2+^‐loaded CaM/CyaA‐ACD is likely to be the predominant conformer in the cell.

**Figure 2:**
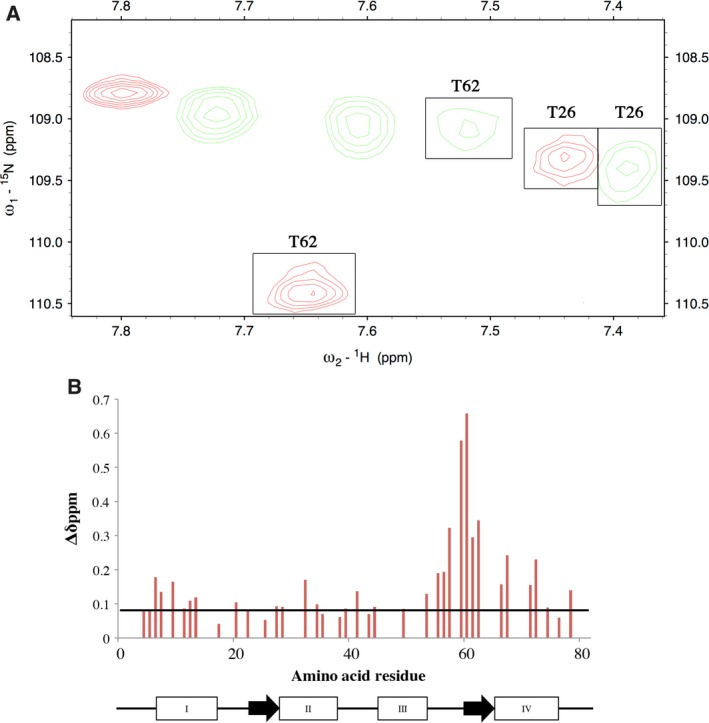
2D ^1^H‐^15^N TROSY spectra demonstrating that the metal binding properties at site II are affected by the presence of CyaA and Ca^2+^ binding in the C terminus of CaM. (A) [^15^N, ^13^C, ^2^H]‐CaM free in the 4Mg^2+^saturation (red) and [^15^N, ^13^C, ^2^H]‐CaM bound to CyaA‐ACD in the presence of 2Mg^2+^/2Ca^2+^ saturation (green). (B) Amide proton‐nitrogen chemical shift differences between 2Mg^2+^/2Ca^2+^‐CaM/CyaA and 4Mg^2+^‐CaM. Selected residues are labeled according to the amino acid residue. The secondary structure of CaM is summarized with rectangles (helices) and arrows (β‐sheets). The average chemical shift change + one standard deviation is indicated by the horizontal black line. The interaction of CyaA‐ACD with the C‐terminal domain of CaM induces chemical shift perturbations at site II in the N‐terminal domain of CaM. It is possible that CyaA‐ACD‐induced conformational modulation of site II might increase Mg^2+^ binding affinity in the N‐terminal domain of CaM.

**Figure 3:**
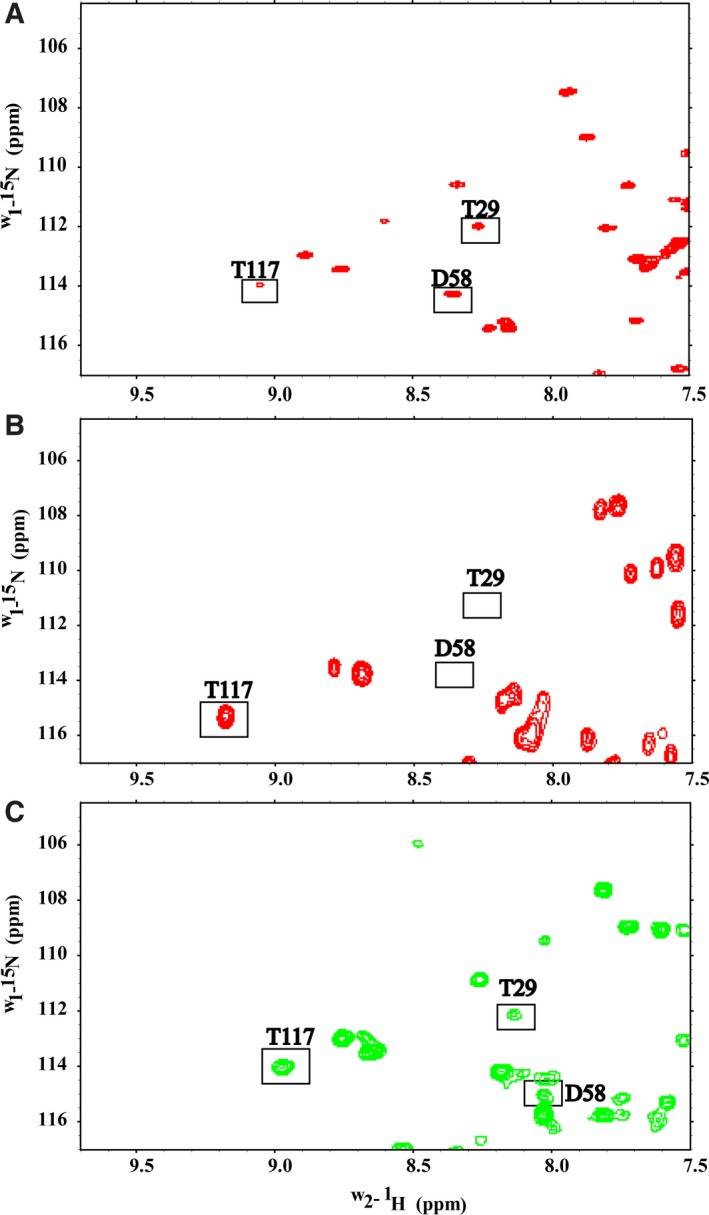
CyaA‐ACD interaction with CaM stabilizes CaM metal binding in the N terminus of CaM. Selected regions of 2D amide proton‐nitrogen correlation spectra are displayed for (A) [^15^N, ^13^C, ^2^H]‐CaM‐free 4Mg^2+^ saturation (red), (B) [^15^N, ^13^C, ^2^H]‐CaM‐free 2Mg^2+^/2Ca^2+^ saturation (dark red), (C) [^15^N, ^13^C, ^2^H]‐CaM/CyaA 2Mg^2+^/2Ca^2+^ saturation (green). The C‐terminal domain of CaM has distinct Mg^2+^‐ and Ca^2+^‐loaded conformations as evidenced by the position of T117 in site III. In the absence of CyaA‐ACD, CaM residues T29 and D58 are broadened in the 2Mg^2+^/2Ca^2+^‐loaded state due to Mg^2+^ exchange. Upon CyaA‐ACD association, the exchange broadening at sites I and II in CaM diminished, which is likely the consequence of increased apparent Mg^2+^ binding affinity induced by CyaA‐ACD association.

CyaA‐ACD binding to CaM not only affects its conformation but it also impacts metal binding properties. We observed that when CyaA‐ACD and Ca^2+^ are bound to the C‐terminal domain, site II of CaM exhibited a structurally unique conformation in the 2Mg^2+^/2Ca^2+^‐loaded state as compared to the free 4Mg^2+^‐CaM (Fig. 2A). Most notably, a more substantial chemical shift difference was observed between 4Mg^2+^‐CaM and 2Mg^2+^/2Ca^2+^‐CaM/CyaA‐ACD for T62 in site II as compared to T26, the corresponding peak in site I. It is possible that CyaA‐ACD association with CaM increases Mg^2+^ binding affinity at site II which results in more pronounced chemical shift perturbations (Fig. 2B). Further evidence supporting that CyaA‐ACD association modulates the metal binding properties of CaM is seen by comparing other regions of NMR spectra of 4Mg^2+^‐CaM, 2Mg^2+^/2Ca^2+^‐CaM, and 2Mg^2+^/2Ca^2+^‐CaM/CyaA‐ACD. The 2D correlation spectra of 4Mg^2+^‐CaM and 2Mg^2+^/2Ca^2+^‐CaM demonstrate that distinct amide proton‐nitrogen chemical shifts are observed in each metal‐bound state (Fig. 3A, B respectively). For example, peaks T29, D58, and T117 mapping to sites I, II, and III, respectively, had chemical shifts which are consistent with CaM being in the 4Mg^2+^‐loaded state (Fig. 3A). In the 2Mg^2+^/2Ca^2+^‐CaM sample, the amide nitrogen of T117 was shifted +1.5 p.p.m. as compared to 4Mg^2+^‐CaM and this position in the spectrum was comparable to that the observed in the Ca^2+^‐loaded state, indicating C‐terminal‐specific Ca^2+^ binding (Fig. 3A, B). However, significant exchange broadening was observed for the amide proton‐nitrogen resonances of T29 and D58 in 2Mg^2+^/2Ca^2+^‐CaM as compared to 4Mg^2+^‐CaM, but no peaks corresponding to Ca^2+^‐loaded protein were observed for any N‐terminal resonances, indicating that Mg^2+^ binding is site‐specific, with conformational exchange occurring between the Mg^2+^‐free and Mg^2+^‐bound states (Fig. 3B). Unfortunately, significant exchange broadening of these N‐terminal resonances in CaM precluded the determination of apparent Mg^2+^ binding affinities by NMR. Unexpectedly, the association of CyaA‐ACD with the C‐terminal domain of CaM reduced Mg^2+^ exchange at sites I and II in the N‐terminal domain as evidenced by the lack of exchanged broadened peaks (Fig. 3C). In the presence of 40 mm Mg^2+^, the amide proton‐nitrogen resonances of T29 and D58 reappeared and were readily detectable in the spectrum of the 2Mg^2+^/2Ca^2+^‐CaM/CyaA‐ACD complex, which suggests that CyaA‐ACD binding to C‐terminal CaM modulates metal exchange most likely by increasing Mg^2+^ binding affinities in the N‐terminal domain. To the best of our knowledge, this is the first report of an allosteric interdomain communication pathway specific to CyaA‐ACD binding that functions in fine‐tuning the Mg^2+^ and Ca^2+^ binding affinities in CaM.

### Global conformational analyses of 4Ca^2+^‐CaM/CyaA‐ACD and 2Mg^2+^/2Ca^2+^‐CaM/CyaA‐ACD complexes

**Figure 4:**
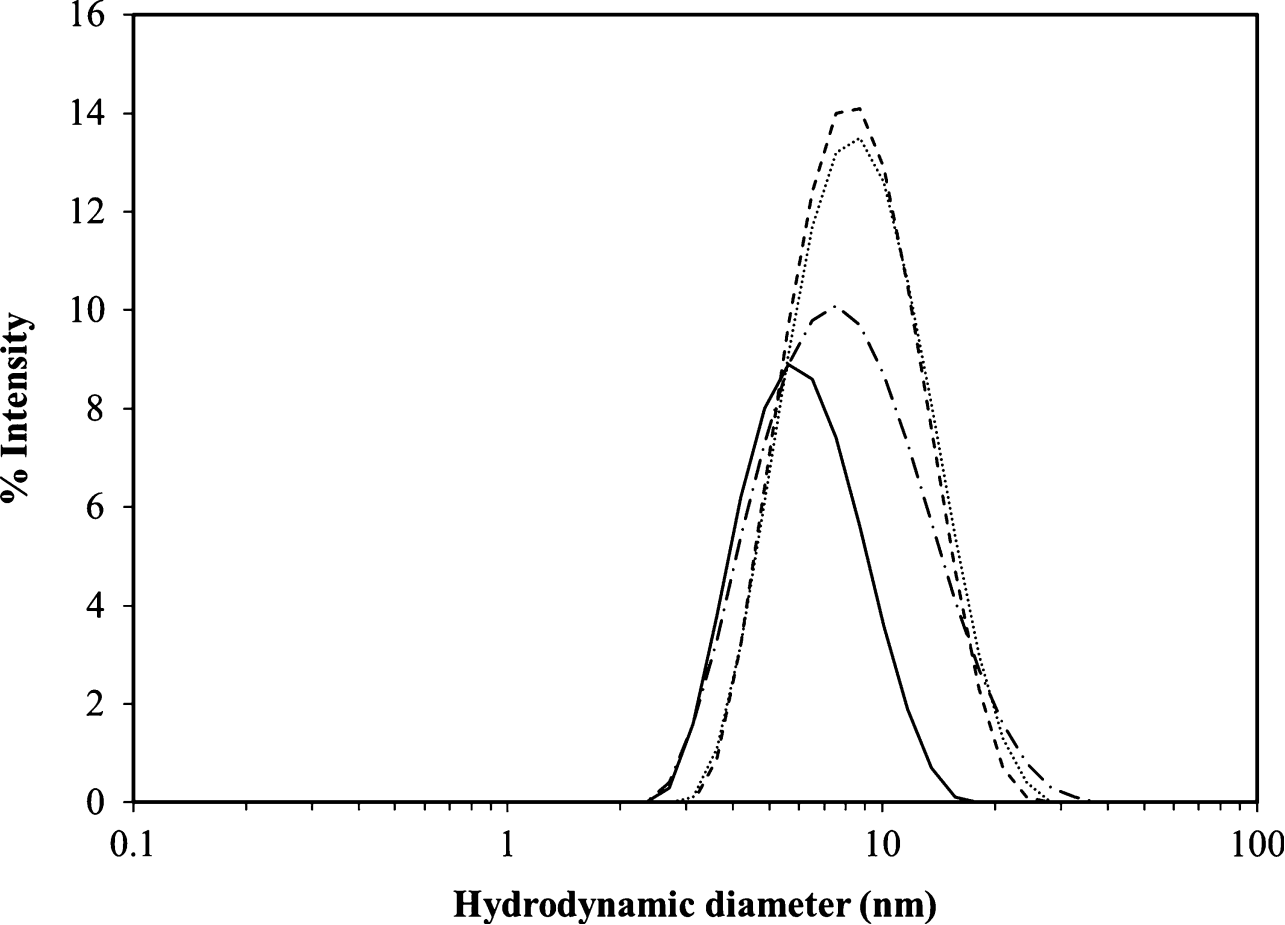
Dynamic light scattering experiments show that the hydrodynamic diameters of CaM/CyaA‐ACD complexes are larger than those measured for free CaM. The intensity versus the hydrodynamic diameter are plotted for free 4Ca^2+^‐CaM (solid black line), 4Ca^2+^‐CaM/CyaA‐ACD (dashed line), 2Mg^2+^/2Ca^2+^‐CaM/CyaA‐ACD (dotted line), and 2Mg^2+^/2Ca^2+^‐CaM(E31D)/CyaA‐ACD (dashed/dotted line). The reduction in the hydrodynamic diameter of 2Mg^2+^/2Ca^2+^‐CaM(E31D)/CyaA‐ACD as compared to 2Mg^2+^/2Ca^2+^‐CaM/CyaA‐ACD provides evidence that this mutant induces a global compaction in this complex.

Dynamic light scaterring has been previously used to examine the global conformation of CaM and CaM/CyaA‐ACD complexes 19 under various conditions. In DLS, the hydrodynamic diameters are determined from the Stokes–Einstein equation based on the presence of a sphere. Measurement of the hydrodynamic diameter of a rod‐shaped protein results in a larger value as compared to that determined for a sphere of similar molecular mass. Previous hydrodynamic studies of the CaM/CyaA‐ACD complex demonstrated that it has an ellipsoid conformation instead of a globular shape in solution 19, 29. In the present study, all samples of CaM and CaM/CyaA‐ACD were determined to be monodisperse and free of aggregation (Fig. 4). The hydrodynamic diameter of 4Ca^2+^‐CaM/CyaA‐ACD increased as compared to free 4Ca^2+^‐CaM (Fig. 4), which indicates the presence of binary complex formation. Comparison of the hydrodynamic diameters of the 4Ca^2+^‐CaM/CyaA‐ACD to 2Mg^2+^/2Ca^2+^‐CaM/CyaA‐ACD, revealed that the metal‐bound complexes have similar global conformations in solution. These findings are consistent with the formation of an elongated CaM/CyaA‐ACD complex 17, 19, 29. While the 4Ca^2+^‐CaM/CyaA‐ACD has been identified as being oblong in solution, this is the first known report of an extended, partially Mg^2+^‐loaded conformation for the CaM/CyaA‐ACD complex.

**Figure 5:**
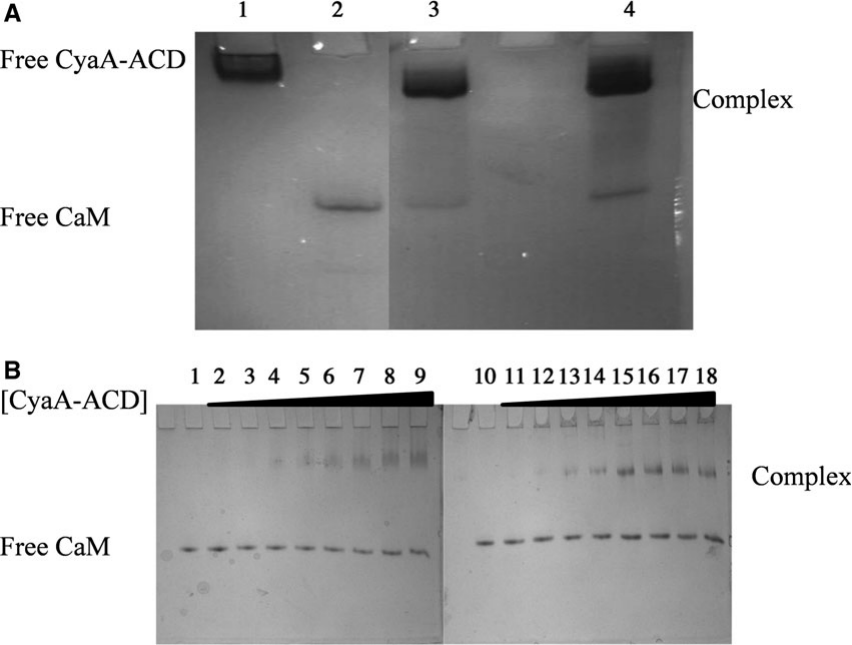
Complex formation between CaM and CyaA‐ACD in the presence of Ca^2+^ or Mg^2+^/Ca^2+^ saturation can be monitored by native PAGE**.** Following SEC, complexes were visualized using native 15% PAGE and Coomassie Brilliant blue staining. **(**A) (Lane 1) CyaA‐ACD, (Lane 2) CaM, (Lane 3) 4Ca^2+^‐CaM/CyaA‐ACD, (Lane 4) 2Mg^2+^/2Ca^2+^‐CaM/CyaA‐ACD. The electrophoretic mobility of complexes consisting of 4Ca^2+^‐CaM/CyaA‐ACD or 2Mg^2+^/2Ca^2+^‐CaM/CyaA‐ACD was intermediate as compared to free CaM and free CyaA. A similar shift in mobility was observed for 4Ca^2+^‐CaM/CyaA‐ACD or 2Mg^2+^/2Ca^2+^‐CaM/CyaA‐ACD suggesting that these complexes have comparable shapes and charges. (B) Titration studies were performed with varying amounts of CyaA‐ACD (0–2.5 μm) added to 5 μm of 4Ca^2+^‐CaM or 2Mg^2+^/2Ca^2+^‐CaM(E31D). (Lane 1) Free 4Ca^2+^‐CaM, (Lanes 2–9) increasing amounts of CyaA‐ACD (Lane 10) Free 2Mg^2+^/2Ca^2+^‐CaM(E31D), (Lanes11–18) increasing amounts of CyaA‐ACD. The mutant 2Mg^2+^/2Ca^2+^‐CaM(E31D)/CyaA‐ACD complex had an increased electrophoretic mobility which indicates it is compacted as compared to the 4Ca^2+^ or 2Mg^2+^/2Ca^2+^‐loaded states.

Native PAGE analyses further supported that the 4Ca^2+^‐CaM/CyaA‐ACD and 2Mg^2+^/2Ca^2+^‐CaM/CyaA‐ACD have comparable global conformations (Fig. 5A). The 4Ca^2+^‐CaM/CyaA‐ACD and 2Mg^2+^/2Ca^2+^ CaM/CyaA‐ACD complexes exhibited similar native PAGE migration patterns, hence, based on previous data demonstrating that 4Ca^2+^‐CaM/CyaA‐ACD is extended, the 2Mg^2+^/2Ca^2+^ CaM/CyaA‐ACD complex is regarded to have an elongated global conformation. These findings are in agreement with reports from Karst *et al*. 29 that CaM/CyaA‐ACD is elongated and that the conformation of free CyaA‐ACD differs from that in the complex. We have demonstrated that CyaA‐ACD interaction with both domains of CaM is necessary to maintain the complex in an extended global state 19. In that same study, we found that disruption of intermolecular contacts between the β‐hairpin of CyaA and CaM decreases the hydrodynamic radius of the complex. Based on these findings, we proposed that mutation of site I in CaM, in particular, modification in side‐chains that are likely to be located at the protein–protein binding interface, would also impact the global conformation of CaM/CyaA in the presence of Ca^2+^ and Mg^2+^ saturation. To test this prediction, we generated CaM(E31D) and examined its metal‐dependent association with CyaA‐ACD.

### Global conformational change in the 2Mg^2+^/2Ca^2+‐^CaM(E31D)/CyaA‐ACD complex

With the CaM(E31D) mutant, our goal was to examine CyaA–ACD interaction using a CaM mutant that was not likely to have substantial conformational changes and was Mg^2+^ specific at site I. The −Z ligand in the metal‐binding loops of canonical EF‐hand proteins, such as CaM and TnC, are Glu acid residues. In Ca^2+^‐loaded CaM, Oε1 and Oε2 of Glu residues coordinate the Ca^2+^ ion in a bidentate fashion. However, in Mg^2+^‐loaded CaM these side‐chain groups do not participate in Mg^2+^ coordination 12, 22. This is in direct contrast to TnC, which has monodentate ligation of the Mg^2+^ ion mediated through Oε1 of Glu in the presence of target peptide 11, 30. TnC has been demonstrated to undergo a compaction in of the metal‐binding loops in the Mg^2+^‐loaded state 11, whereas CaM does not coordinate Mg^2+^ through the −Z ligand, so metal ligation is limited to the N‐terminal portion of the loop 12, 22. Metal binding specificity can be controlled by the amino acid side‐chain present at position 12 of the EF‐hand loop. In other EF‐hand proteins, mutation of the Glu residue at the −Z position to Asp has been shown to promote Mg^2+^ binding specificity without inducing substantial conformational changes in the proteins 31, 32.

In this study, CaM(E31D) was engineered in order to optimize the Mg^2+^ binding properties at site I, without invoking a charge change at the −Z ligand, so that we would minimize the possibility of disrupting intermolecular association with CyaA‐ACD. We used DLS and native PAGE analyses to assess the structural consequences of mutant CaM(E31D) on CyaA‐ACD association. We expect CaM(E31D) to be Mg^2+^ specific at site I while site II is capable of Mg^2+^ or Ca^2+^ coordination. Surprisingly, we observed that mutation at this position increased the degree of compaction in 2Mg^2+^/2Ca^2+^‐CaM(E31D)/CyaA‐ACD, which results in a reduction in the hydrodynamic diameter from 9 nm in the 2Mg^2+^/2Ca^2+^‐CaM/CyaA‐ACD complex to 7.5 nm in the mutant 2Mg^2+^/2Ca^2+^‐CaM(E31D)/CyaA‐ACD complex (Fig. 4). Similarly, the electrophoretic mobility of the 2Mg^2+^/2Ca^2+^‐CaM(E31D)/CyaA‐ACD complex was altered as compared to 4Ca^2+^CaM/CyaA‐ACD or 2Mg^2+^/2Ca^2+^CaM/CyaA‐ACD, which supports that this mutant complex most likely has a different global shape (Fig. 5B). Taken together, these data suggest that alteration of the −Z ligand disrupts the intermolecular associations needed for formation of an extended Mg^2+^‐loaded CaM/CyaA‐ACD complex. Previously, we reported the existence of intermolecular association between site I in CaM and CyaA‐ACD that contributes to the extended global conformation 19. Based on the observations in our current work, we concluded that site I mutation results in the loss of stabilizing interactions between N‐terminal CaM and CyaA‐ACD in the 2Mg^2+^/2Ca^2+^‐CaM/CyaA‐ACD complex. These data point to a model of CaM‐dependent activation of CyaA‐ACD that requires an extended complex conformation similar to that observed for *B*. *anthracis* EF 14. It is interesting to speculate that this extended conformation mechanism in CaM/CyaA‐ACD might also be one that permits the modification of metal binding in CaM. While the role of CyaA‐ACD binding in the modulation of CaM's metal binding properties remains unclear, it is likely to contribute to enzyme activation by desensitizing the system to fluctuations in intracellular Ca^2+^ levels.

In summary, comparing 4Ca^2+^‐CaM/CyaA‐ACD to 2Mg^2+^/2Ca^2+^‐CaM/CyaA‐ACD reveals that Mg^2+^ binding is localized to sites I and II of N‐terminal CaM. Interaction with CyaA‐ACD increases the apparent Ca^2+^ binding in C‐terminal CaM as evidenced by prohibited metal exchange in the CaM/CyaA‐ACD complex. The presence of CyaA‐ACD and Ca^2+^ binding to C‐CaM modulates site II of Mg^2+^‐CaM. In the absence of an effector, N‐terminal CaM residues are prone to broadening, however, the presence of CyaA‐ACD reduces Mg^2+^ exchange in N‐terminal CaM. DLS data show similarities between 4Ca^2+^‐CaM/CyaA‐ACD and 2Mg^2+^/2Ca^2+^‐CaM/CyaA‐ACD global conformations of these complexes. In comparison to other prokaryotic adenylate cyclase toxins, such as EF from *Bacillus anthracis* and ExoY from *Pseudomonas aeruginosa*, there is only 25% sequence identity at the amino acid level with respect to the ATP‐binding domain. While the host cell activator is unknown for ExoY, EF and CyaA‐ACD are both CaM‐dependent and have evolved molecular mechanisms for activation that are distinct from eukaryotic adenylate cyclases. Taken together, these data indicate that CyaA‐ACD binding likely modulates metal binding to CaM. The molecular role CyaA‐ACD plays in fine‐tuning CaM's metal binding capabilities remains to be determined. High‐resolution structures of the CaM/CyaA‐ACD complexes in the Ca^2+^‐ and Mg^2+^‐loaded states are necessary in order to understand the structural mechanisms by which of prokaryotic adenylate cyclase toxins are controlled.

## Author contributions

TIS, CCE, CWJ, and NLF performed the experiments, analyzed data, and interpreted results. TIS and NLF wrote the manuscript. All authors provided comments and edits in the development of the manuscript.
